# *Levilactobacillus brevis* MG5311 Alleviates Ethanol-Induced Liver Injury by Suppressing Hepatic Oxidative Stress in C57BL/6 Mice

**DOI:** 10.3390/microorganisms10122488

**Published:** 2022-12-15

**Authors:** Hyunna Jung, Sohyeon You, Soo-Im Choi, Chang-Ho Kang, Gun-Hee Kim

**Affiliations:** 1 Department of Bio-Health Convergence, Duksung Women’s University, Seoul 01369, Republic of Korea; 2 MEDIOGEN, Co., Ltd., Biovalley 1-ro, Jecheon-si 27159, Republic of Korea; 3 Department of Food and Nutrition, Duksung Women’s University, Seoul 01369, Republic of Korea

**Keywords:** ethanol, liver injury, *Levilactobacillus brevis*, oxidative stress, lipid peroxidation

## Abstract

Alcoholic liver disease (ALD), caused by excessive alcohol consumption, leads to high mortality. We investigated the hepatoprotective effect of *Levilactobacillus brevis* MG5311 in C57BL/6 mice with liver injuries induced by chronic ethanol plus binge feeding. *L. brevis* MG5311 was administered orally at a dose of 1 × 10^9^ CFU/mouse once daily for 32 days. *L. brevis* MG5311 administration significantly reduced serum ALT, AST, and triglyceride (TG) levels in ethanol-fed mice. *L. brevis* MG5311 also decreased malondialdehyde levels and increased glutathione peroxidase (GPx) activity in liver tissues. In addition, hepatic TG content and histopathological scores were significantly reduced. *L. brevis* MG5311 increased the protein expression of SIRT1, PPARα, SOD1, CAT, and GPx 1/2 in liver tissue, while inhibiting CYP2E1 and SREBP-1c. These results indicated that *L. brevis* MG5311 alleviated ethanol-induced liver injury by inhibiting hepatic oxidative stress and promoting lipid metabolism. Therefore, *L. brevis* MG5311 may be a useful probiotic candidate for ameliorating or preventing ALD.

## 1. Introduction

Alcoholic injury, characterized by multi-systemic and diverse pathophysiology, adversely affects a patient’s quality of life. Alcoholic liver disease (ALD) is caused by the chronic excessive consumption of alcohol. The number of ALD patients is rapidly increasing worldwide, and the associated high morbidity and mortality rates are emerging as serious issues [1,2]. ALD covers a broad spectrum of diseases, including early-stage fatty liver, steatohepatitis, liver fibrosis, cirrhosis, and liver cancer [3]. Metabolic disorders, such as obesity and diabetes, may exacerbate liver problems caused by the fatty liver disease along with ALD [4,5]. However, there is currently no approved drug or appropriate treatment other than abstinence or liver transplantation [6]. Therefore, there is an urgent need to develop new therapeutic agents to prevent or alleviate ALD.

Excessive alcohol consumption induces endotoxemia, alters the gut microbiome profile (dysbiosis), disrupts the gut barrier function, and increases intestinal permeability. Metabolites such as lipopolysaccharides (LPS) produced by gut bacteria leak into the hepatic portal vein, causing features of ALD, such as liver inflammation and fibrosis [7,8]. Alcohol metabolism in the liver promotes the production of acetaldehydes and reactive oxygen species (ROS) and weakens the antioxidant defense system. Hepatic oxidative stress results in alcohol-induced lipid peroxidation and tissue damage, including metabolic changes, inflammation, and cell membrane dysfunction [9].

The World Health Organization (WHO) defines probiotics as “living microorganisms that, when consumed in adequate amounts, confer a health benefit on the host,” and the most commonly used as probiotics are lactic acid bacteria (LAB) [10]. Some LAB strains provide various health benefits to the host, such as antimicrobial, anti-inflammatory, antioxidative, anti-allergic, and improving lipid metabolism and immune responses [11,12,13,14]. Recent studies have extensively demonstrated a close relationship between host health and gut microbiota [15]. The “gut-liver axis” has demonstrated that the imbalance of the gut microflora is an important factor exacerbating the progression of ALD [16]. LAB can prevent ALD by inhibiting hepatic oxidative stress and improving intestinal barrier function, thereby reducing endotoxemia in the gut-liver axis [16,17]. Segawa et al. (2008) reported that heat-killed *Lactobacillus brevis* SBC8803 inhibited gut-derived endotoxin migration into the liver of ethanol-containing diet-fed mice [18]. *Lactobacillus rhamnosus* GG ameliorated chronic alcohol-induced liver and intestinal barrier injury [19]. However, the mechanism by which LAB prevents ALD progression remains unclear.

In a previous study, we isolated and identified *Levilactobacillus brevis* MG5311 from fermented food. We confirmed that this strain protects against ethanol-induced HepG2 cell damage by increasing aldehyde dehydrogenase (ALDH) activity, regulating cytochrome P450 2E1 (CYP2E1), activating antioxidant enzymes, and modulating lipid metabolism. In addition, this strain has been confirmed to be safe and stable owing to its resistance to the gastrointestinal tract and adhesion ability in vitro [20]. Therefore, this study aimed to determine whether *L. brevis* MG5311 has a preventive effect on ethanol-induced liver injury in mice and to identify the mechanisms of action, thereby confirming its potential as a useful new probiotic candidate for preventing or improving ALD.

## 2. Materials and Methods

### 2.1. Materials

Lieber-DeCarli regular control liquid diets (D710027) and Lieber-DeCarli regular ethanol liquid diets (D710260) were purchased from Dyets Inc. (Bethlehem, PA, USA). Maltose dextrin was purchased from BioServ (Flemington, NJ, USA). Nonidet^TM^ P 40 substitute and 200-proof ethyl alcohol were purchased from Sigma-Aldrich (Saint Louis, MO, USA). Antibody against Cytochrome P450 2E1 (CYP2E1) was purchased from Invitrogen (Waltham, MA, USA). Antibodies against superoxide dismutase (SOD), catalase (CAT), AMP-activated kinase (AMPK), and GAPDH were purchased from Cell Signaling Technology (Danvers, MA, USA). Antibodies against aldehyde dehydrogenase 2 (ALDH2), sirtuin 1 (SIRT1), sterol regulatory element-binding protein 1c (SREBP-1c), peroxisome proliferator-activated receptor α (PPARα), and glutathione peroxidase 1/2 (GPx 1/2) were purchased from Santa Cruz Biotechnology (Dallas, TX, USA). Malondialdehyde (MDA, ab118970), GPx (ab102530), and triglyceride (TG, ab65336) assay kits were purchased from Abcam (Cambridge, UK).

### 2.2. L. brevis MG5311 Cultivation

*L. brevis* MG5311, isolated from food, was supplied by MEDIOGEN Co., Ltd. (Jecheon, Korea). The GenBank accession number for the 16S rRNA gene sequence of *L. brevis* MG5311 is MN720513.1, as assessed by the National Center of Biotechnology Information (NCBI). The strain was activated by culturing in de Man, Rogosa, and Sharpe broth (MRS, Difco, Detroit, MI, USA) medium at 37 °C for 18 h. Cells were collected by centrifugation (15,000× *g*, 15 min, 4 °C), and the harvested pellets were mixed with the cryoprotectant cocktail and then freeze-dried [21].

### 2.3. Animal Experimental Procedure

C57BL/6J mice (male, 7 weeks old, and 20–22 g) were obtained from the Central Lab. Animal Inc. (Seoul, Korea). Mice were housed in one mouse per cage in an air-conditioned room at 23 ± 1.0 °C and a relative humidity of 45 ± 5% under a 12 h light/dark cycle. Animal experiments were approved by the Institutional Animal Care and Use Committee of Duksung Women’s University (No. 2021-005-006). All efforts were made to minimize the pain in the mice.

Mice were randomly divided into three groups (*n* = 6/group). (1) normal diet (ND), (2) ethanol diet (EtOH), and (3) ethanol diet plus *L. brevis* MG5311 (MG5311). Ethanol-induced liver injury was induced in mice for 32 days. The gavage volume and concentration of binge feeding were determined according to the NIAAA protocol [22]. Briefly, all mice were acclimatized to a liquid diet for 4 days using the Lieber-DeCarli control diet. On day 5, the ethanol-diet-fed groups were fed a Lieber-DeCarli-ethanol diet containing 0.75% ethanol for 3 days. Afterward, mice were fed the diet containing 1.5% ethanol for 3 days and the diet containing 3.75% ethanol for 4 days sequentially. The mice were then fed a diet containing 5% ethanol for 22 days. The diets were served daily at 3 p.m. Finally, on day 33, 31.5% (*v/v*) ethanol solution was orally binged to the ethanol-diet-fed groups. The control diet group was fed a 45% (*w/v*) maltose dextrin solution instead of ethanol. MG5311 strain powder was dissolved in sterile phosphate-buffered saline (PBS) and orally administered once a day at 1 × 10^9^ CFU/mouse during feeding of the ethanol-containing diet. Nine hours after binge feeding, all mice were anesthetized by CO_2_ inhalation and euthanized by cardiac puncture.

### 2.4. Measurement of Body and Liver Tissue Weight and Food Intake

After the liquid diet adaptation was completed, the mice were weighed every 3 days for the experimental period. Food intake was measured daily. Liver tissues dissected from mice were washed with PBS buffer and immediately weighed. Liver tissues were stored at −70 °C until further use. The weight ratio was expressed as the liver tissue weight compared to the last body weight measurement before the mice were sacrificed.

### 2.5. Biochemical Analysis

All blood samples were collected into EDTA-coated tubes and placed for 30 min at room temperature. The serum was obtained by centrifugation at 4000 rpm for 20 min at 4 °C. Serum alanine aminotransferase (ALT), aspartate transaminase (AST), and TG levels were measured using an auto-serum analyzer (FUJI, Tokyo, Japan).

### 2.6. Measurement of MDA Concentration

Liver tissue (10 mg) was homogenized in a lysis buffer with butylated hydroxytoluene (BHT). The tissue lysate was then centrifuged at 13,000× *g* for 10 min. The supernatant was then collected for further analysis. The MDA concentration in the liver tissue was measured using a commercial kit according to the manufacturer’s instructions [23].

### 2.7. Measurement of GPx Activity

Liver tissue (100 mg) was homogenized in 200 μL assay buffer. The homogenate was centrifuged at 10,000× *g* for 15 min at 4 °C. The supernatant was harvested, and 10-fold diluted samples were used for analysis. GPx activity in the liver tissue was measured using a commercial kit according to the manufacturer’s instructions [24].

### 2.8. Measurement of TG Contents

To measure TG content, liver tissue (100 mg) was homogenized in 1 mL of 5% Nonidet-P40 substitute in distilled water. [25]. The homogenate was heated at 90 °C for 3 min in a water bath and cooled at room temperature, which was repeated twice to solubilize all the TG. The homogenate was then centrifuged at 12,500 rpm for 2 min. The supernatant was harvested, and 20-fold diluted samples were used for analysis. TG content in the liver tissue was measured using a commercial kit (Abcam, Cambridge, UK) according to the manufacturer’s instructions. TG levels were quantified based on the protein concentration of the extract using the Bradford protein assay (Bio-Rad, Hercules, CA, USA).

### 2.9. Histopathological Examination

Liver tissues were fixed in 10% formalin solution for 24 h and embedded in paraffin to analyze histopathological changes. Subsequently, 4-μm liver sections were stained with hematoxylin and eosin (H&E) solution. Histopathological scores for hepatic steatosis and inflammation were quantified and expressed simply by the summation of 4 individual grades as follows: 0 (<5%), 1 (5–33%), 2 (33–66%), and 3 (>66%) [26]. A board-certified toxicological pathologist blindly performed all histological evaluation procedures.

### 2.10. Western Blot Analysis

The expression levels of signaling proteins involved in alcohol-induced mechanisms in liver tissue were determined using Western blot analysis. Briefly, the liver tissues were homogenized in PRO-PREP™ protein extraction solution (iNtRON Biotechnology Co., Seongnam, Korea) containing a protease and phosphatase inhibitor cocktail (GenDEPOT, Katy, TX, USA). Homogenized samples were centrifuged at 12,000 rpm for 20 min at 4 °C, and the supernatant was collected. Protein levels in the lysates were quantified using Bradford assay. The proteins (10 μg) were mixed with a loading buffer, electrophoresed on a 10% sodium dodecyl sulfate-polyacrylamide (SDS) gel, and transferred to a polyvinylidene difluoride (PVDF) membrane for 3 h at 70V. Then, the proteins were blocked with 10% skim milk in Tris-buffered saline containing Tween-20 (TBST) for 60 min, and the membranes were incubated overnight with primary antibodies against ALDH2, CYP2E1, SIRT1, SREBP-1c, PPARα, SOD, CAT, GPx 1/2, and GAPDH (1:1000) in TBST at 4 °C. The membranes were washed three times with TBST buffer. Membranes were then incubated with goat anti-rabbit IgG-HRP or goat anti-mouse IgG-HRP conjugated secondary antibodies (1:4000, diluted with 5% skim milk in TBST) for 60 min at room temperature and washed three times with TBST buffer. Proteins were visualized using an ECL detection kit (Amersham Pharmacia, Piscataway, NJ, USA) and quantified using Image J program 1.3 (National Institute of Health, Bethesda, MD, USA).

### 2.11. Statistical Analysis

All data are presented as the mean ± standard deviation. Statistical analyses were performed using Prism 5 (GraphPad Software Inc., La Jolla, CA, USA). One-way analysis of variance (ANOVA) was used to compare the mean value between groups. Significant differences between the groups were evaluated by Dunnett’s Multiple Comparison Test (*p* < 0.05).

## 3. Results

### 3.1. Effect of L. brevis MG5311 on Body and Liver Weight and Food Intake

The chronic binge ethanol-feeding animal model is easy to perform and closely reproduces acute-on-chronic liver injury in patients with ALD. However, extensive steatosis and elevated serum ALT or AST levels induced by multiple chronic binge drinking also increase animal mortality [27].

**Figure 1:**
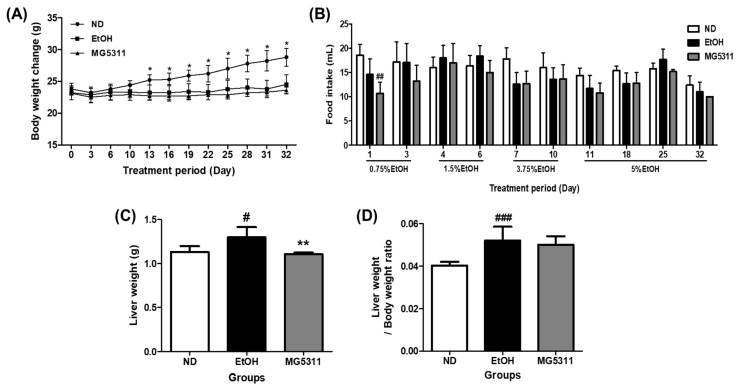
Effect of *L. brevis* MG5311 on (**A**) body weight change, (**B**) food intake, (**C**) liver weight, and (**D**) liver/body weight ratio in chronic ethanol-fed mice. Mice were fed with ND or EtOH diets for 32 days and binge drinking once on day 33. EtOH-diet mice were treated with an experimental vehicle or MG5311 strain. Data are presented as mean ± SD (*n* = 6). Significance was analyzed by Dunnett’s test. ^#^ *p* < 0.05, ^##^ *p* < 0.01, ^###^ *p* < 0.001, vs. ND group. * *p* < 0.05, ** *p* < 0.01 vs. EtOH group. ND; normal diet, EtOH; ethanol diet, MG5311; ethanol diet-*L. brevis* MG5311.

To induce alcoholic liver injury, mice were fed 0.75 to 5% ethanol for 32 days and received one binge dose of 31.5% (*vol/vol*) ethanol according to the NIAAA model [20]. The body weights of the EtOH-fed groups increased slightly throughout the experiment but were not significant. In contrast, the ND group showed a significant increase after 13 days (*p* < 0.05). However, no significant difference was observed between the EtOH and MG5311 groups (Figure 1A). In addition, there was no significant difference in food intake between the experimental groups (Figure 1B). However, the weight loss and difficulty in recovering in ethanol-fed mice may be due to inefficient energy use and reduced nutrient intake by chronic alcohol intake [28].

In addition, the weight of liver tissue and liver/body weight ratio significantly increased in the EtOH-fed group compared to the ND group (Figure 1C,D).

### 3.2. Effect of L. brevis MG5311 on Changes in Serum Biomarkers

ALT and AST are important metabolic enzymes in hepatocytes and are generally present at low levels in plasma. ALT, AST, and TG leak into the blood in hepatocytes as liver damage caused by toxic substances progress [29]. In this study, serum obtained from all mice was analyzed to confirm the hematological changes caused by ethanol intake. The EtOH-fed group showed significantly increased AST and ALT levels compared to those in the ND group, indicating that alcohol can damage plasma and hepatocytes. In contrast, ALT and AST levels in the MG5311-treated group significantly decreased by 34.7% and 40.2%, respectively, compared to those in the EtOH-fed group (*p* < 0.05).

**Figure 2:**
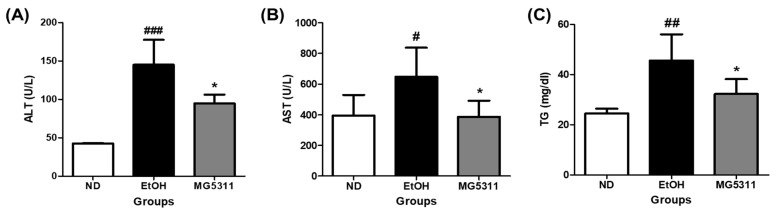
Effect of *L. brevis* MG5311 on hepatic markers in the serum of ethanol-fed mice. (**A**) alanine transferase (ALT); (**B**) alanine aminotransferase (AST); and (**C**) triglyceride (TG). Data are presented as mean ± SD (*n* = 6). Significance was analyzed by Dunnett’s test. ^#^ *p* < 0.05, ^##^ *p* < 0.01, ^###^ *p* < 0.001 vs. ND group. * *p* < 0.05 vs. EtOH group. ND; normal diet, EtOH; ethanol diet, MG5311; ethanol diet-*L. brevis* MG5311.

TG metabolism in liver tissue and blood TG levels are highly correlated [30]. In this study, the TG level in the serum of the MG5311 group (32.25 ± 5.91 mg/dL) was significantly lower than that in the EtOH-fed group (45.60 ± 10.43 mg/dL) (Figure 2). These results showed that MG5311 administration suppressed the elevation of AST, ALT, and TG levels by ethanol intake in mice.

### 3.3. Effect of L. brevis MG5311 on the Protein Expression of ALDH2 and CYP2E1

**Figure 3:**
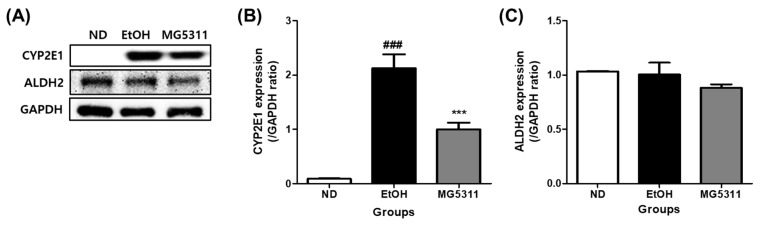
Effect of *L. brevis* MG5311 on protein expression of ethanol metabolism enzymes in liver tissues of ethanol-fed mice. (**A**) representative Western blot; (**B**) cytochrome P450 2E1 (CYP2E1); and (**C**) aldehyde dehydrogenase 2 (ALDH2). Data are presented as mean ± SD (*n* = 3). Significance was analyzed by Dunnett’s test. ^###^ *p* < 0.001 vs. ND group. *** *p* < 0.001 vs. EtOH group. ND; normal diet, EtOH; ethanol diet, MG5311; ethanol diet-*L. brevis* MG5311.

The deleterious effects of alcohol metabolism primarily occur in hepatocytes [31]. Alcohol is primarily oxidized to acetic acid by the oxidative enzymes ADH and ALDH in the liver [32]. CYP2E1 is abundantly expressed in hepatocytes and is the most crucial enzyme in the hepatic microsomal alcohol oxidation system (MEOS) in binge drinking [33]. In this study, the protein expression of CYP2E1 and ALDH2 was evaluated to confirm the effect of MG5311 on alcohol metabolism in the liver using Western blotting. The EtOH-fed group showed significantly upregulated CYP2E1 expression compared to the ND group (*p* < 0.001). In contrast, CYP2E1 expression in the MG5311 group was 0.5-fold lower than in the EtOH group (*p* < 0.001) (Figure 3A,B). However, there was no significant difference in the expression level of ALDH2 between the groups (Figure 3A,C).

### 3.4. Effect of L. brevis MG5311 on MDA Content and GPx Activity in Liver Tissue

**Figure 4:**
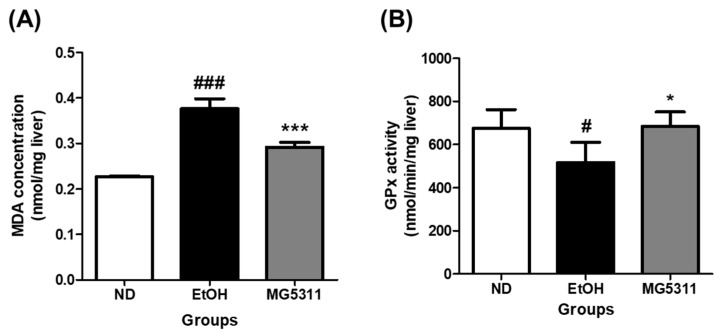
Effect of *L. brevis* MG5311 on (**A**) malondialdehyde (MDA) concentration; and (**B**) glutathione peroxidase (GPx) activity in liver tissue of ethanol-fed mice. Data are presented as mean ± SD (*n* = 3). Significance was analyzed by Dunnett’s test. ^#^ *p* < 0.05, ^###^ *p* < 0.001 vs. ND group. * *p* < 0.05, *** *p* < 0.001 vs. EtOH group. ND, normal diet group; EtOH, ethanol diet group; MG5311, ethanol diet-*L. brevis* MG5311 administered group.

Lipid peroxidation mediated by alcohol exposure increases dramatically within liver tissue and is a cause of liver injury [31]. We measured the levels of MDA and the activity of the endogenous scavenger GPx in hepatic tissues to determine the effect of *L. brevis* MG5311 on inhibiting lipid peroxidation. MDA levels were significantly increased in EtOH-fed groups compared to the ND group (*p* < 0.001), indicating that lipid peroxidation was induced by ethanol administration. However, the MDA level in the MG5311 group was 0.78-fold lower than that in the EtOH group (*p* < 0.001) (Figure 4A).

GPx is a family of enzymes that constitute the primary antioxidant defense system [34]. In this study, the GPx activity of the EtOH-fed group decreased by 23.4% compared with that of the ND group (*p* < 0.05). On the other hand, GPx activity in the MG5311 group increased by 32.4% compared to that in the EtOH group (*p* < 0.05) (Figure 4B). Therefore, these results confirmed that MG5311 administration plays a role in ameliorating ethanol-induced liver damage by lowering MDA levels and increasing GPx activity as a defense system in liver tissues.

### 3.5. Effect of L. brevis MG5311 on the Protein Expression of Antioxidant Enzymes

**Figure 5:**
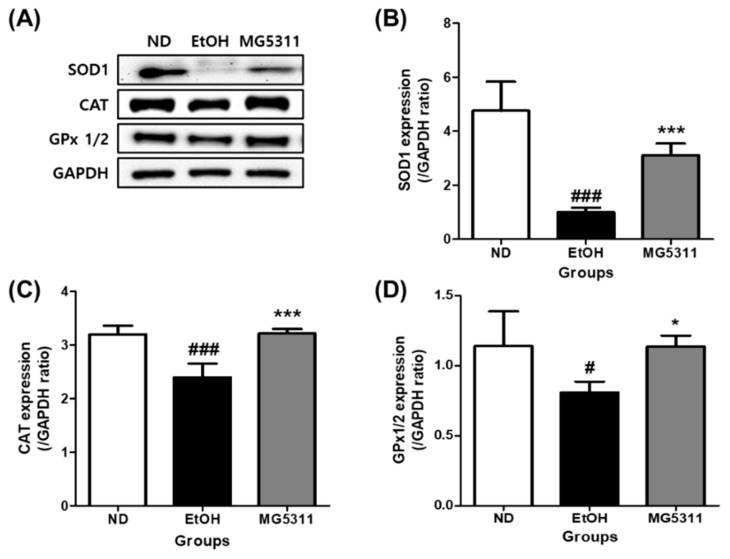
Effect of *L. brevis* MG5311 on protein expression of antioxidant enzymes in the liver tissue of ethanol-fed mice. (**A**) representative Western blot; (**B**) superoxide dismutase 1 (SOD1); (**C**) catalase (CAT); and (**D**) glutathione peroxidase 1/2 (GPx 1/2). Data are presented as mean ± SD (*n* = 3). Significance was analyzed by Dunnett’s test. ^#^ *p* < 0.05, ^###^ *p* < 0.001 vs. ND group. * *p* < 0.05, *** *p* < 0.001 vs. EtOH group. ND, normal diet group; EtOH, ethanol diet group; MG5311, ethanol diet-*L. brevis* MG5311 administered group.

Alcohol causes hepatic oxidative stress and weakens antioxidant enzyme activity [35]. Therefore, restoring the activity of antioxidant enzymes such as SOD, CAT, and GPx-1 alleviates ALD. We measured the protein expression levels of antioxidant enzymes in the liver tissues. The expression levels of both Cu/Zn-SOD (SOD1) and CAT were significantly lowered in the EtOH-fed group than in the ND group (*p* < 0.001). However, the expression levels of both SOD1 and CAT in the MG5311 administered group was 3.1-fold (*p* < 0.001) and 1.3-fold (*p* < 0.001) higher than those in the EtOH group (Figure 5). In addition, the GPx 1/2 expression levels were significantly higher than those in the EtOH group (*p* < 0.05).

### 3.6. Effect of L. brevis MG5311 on TG Contents and Histological Changes in Liver Tissue

**Figure 6:**
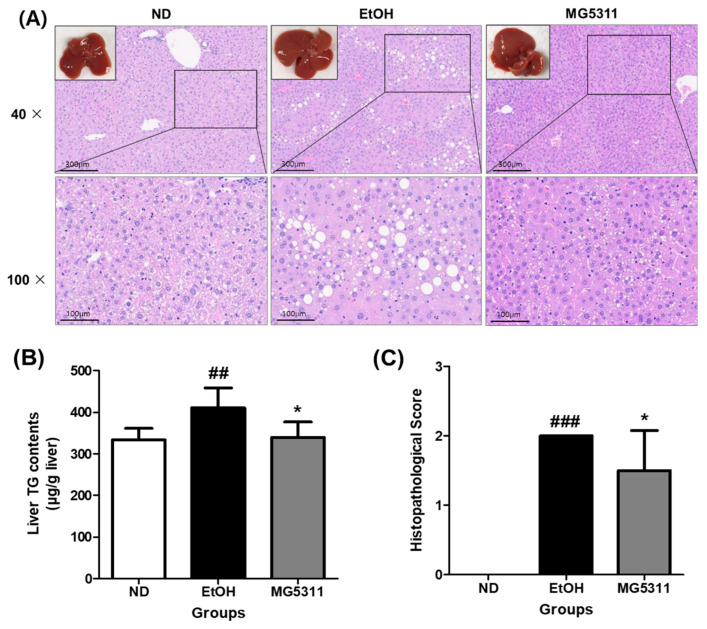
Effect of *L. brevis* MG5311 on histological changes in liver tissues of ethanol-fed mice. (**A**) representative images of liver tissue and H&E-stained liver tissue (40× and 100× magnification); (**B**) hepatic TG content; and (**C**) histopathological score. Data are presented as mean ± SD (*n* = 6). Significance was analyzed by Dunnett’s test. ^##^ *p* < 0.01, ^###^ *p* < 0.001 vs. ND group. * *p* < 0.05 vs. EtOH group. ND, normal diet group; EtOH, ethanol diet group; MG5311, ethanol diet-*L. brevis* MG5311 administered group.

Hepatic steatosis is the first consequence of alcohol abuse and is characterized by the excessive accumulation of TG in hepatocytes due to impaired lipid metabolism [31]. The histopathological features of the liver tissues were assessed using H&E staining. The ND group showed an increase in body weight during the experiment, but no steatosis was observed in the liver tissues. Micro-vesicular steatosis and inflammation were significantly higher in the EtOH-fed group than in the ND group (*p* < 0.001). However, the MG5311 group showed lower steatosis and inflammation levels than the EtOH group (*p* < 0.05) (Figure 6A,C).

The TG content in the liver tissues was quantified using a commercial kit. In the EtOH-fed group (410.45 ± 47.76 μg/g liver), the TG content was significantly higher than that in the ND group (*p* < 0.01). In contrast, the TG levels in the MG5311 group (339.06 ± 37.49 μg/g liver) were considerably lower than those in the EtOH group (*p* < 0.05) (Figure 6B).

### 3.7. Effect of L. brevis MG5311 on Protein Expression of Hepatic Steatosis-Related Genes

**Figure 7:**
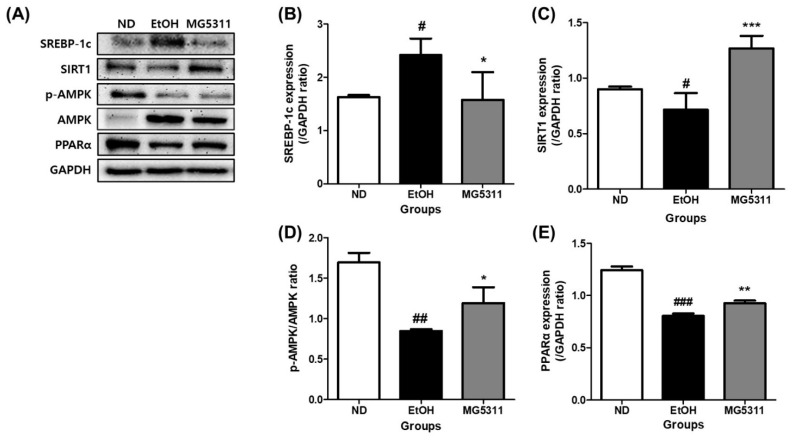
Effect of *L. brevis* MG5311 on hepatic steatosis-related gene expression in liver tissues of ethanol-fed mice. (**A**) representative Western blot; (**B**) sterol regulatory element-binding transcription factor 1c (SREBP-1c); (**C**) sirtuin 1 (SIRT1); (**D**) phospho-AMP-activated protein kinase/AMP-activated protein kinase (p-AMPK/AMPK) (**E**) peroxisome proliferator-activated receptor α (PPARα). Data are presented as mean ± SD (*n* = 3). Significance was analyzed by Dunnett’s test. ^#^ *p* < 0.05, ^##^ *p* < 0.01, ^###^ *p* < 0.001 vs. ND group. * *p* < 0.05, ** *p* < 0.01, *** *p* < 0.001 vs. EtOH group. ND, normal diet group; EtOH, ethanol diet group; MG5311, ethanol diet-*L. brevis* MG5311 administered group.

Alcohol consumption increases the fat synthesis and reduces fatty acid oxidation, leading to metabolic disorders and excessive lipid accumulation [36]. We investigated the protein expression levels of hepatic steatosis-related genes in ethanol-fed mice. The EtOH-fed group showed significantly downregulated SIRT1 and PPARα expression compared with the ND group. However, the expression levels of both genes in the MG5311 group were 1.8-fold (*p* < 0.001) and 1.2-fold (*p* < 0.01) higher than those in the EtOH group (Figure 7B,D).

In addition, the expression level of SREBP-1c was higher in the EtOH group than in the ND group (*p* < 0.05) (Figure 7C). However, MG5311 administration significantly lowered SREBP-1c expression (*p* < 0.05). AMPK phosphorylation was not observed in EtOH-fed groups.

## 4. Discussion

ALD is a complex disease that involves several pathogenic metabolic mechanisms. ALD is caused by oxidative stress induced by alcohol metabolism, mutagenic acetaldehyde formation, and pro-inflammatory cytokine production [37]. Therefore, functional supplements that exhibit antioxidant activity and modulate lipid metabolism in the liver may serve as therapeutic agents to prevent ALD. Studies have demonstrated that probiotics alleviate alcohol-induced liver damage via antioxidant and anti-inflammatory activity [38,39,40,41]. Therefore, the present study investigated the hepatoprotective effects and mechanisms of the probiotic candidate *L. brevis* MG5311 administration in chronic and binge ethanol-fed mice.

Alcohol metabolism increases the production of acetaldehyde, which causes protein inactivation and DNA damage [32]. ALDH plays an essential role in converting the highly toxic acetaldehyde decomposed by ADH into acetic acid and oxidizing acetic acid to carbon dioxide and water. In addition, the conversion of aldehyde to innocuous acetic acid by ALDH suggests a role in primary protection against ROS [42]. ALDH2 is the main enzyme involved in alcohol-derived acetaldehyde metabolism [43]. In a previous study, *L. plantarum* HFY09 improved ALDH levels in the liver of ethanol-fed mice [44]. *L. brevis* HY7410 lowers blood alcohol levels by enhancing ADH and ALDH activity [45]. Our previous in vitro study confirmed that cell-free extracts of MG5311 increase ALDH activity in ethanol-induced HepG2 cells [18]. However, it was not confirmed that MG5311 administration did not affect ALDH2 protein expression in the liver of ethanol-treated mice.

Binge drinking metabolism involves the induction of CYP2E1 in the microsomal system and is a major cause of ALD [33]. CYP2E1 in alcohol metabolism generates ROS, which causes various types of tissue damage, such as oxidative stress, metabolic changes, and membrane dysfunction of intracellular organelles [46]. Upregulated intestinal CYP2E1 increases nitroxidative stress and promotes gut leakage, which may lead to inflammatory liver damage and endotoxemia [47]. *L. rhamnosus* GG supplementation decreased CYP2E1 expression and TNFα production [41]. This study confirmed that *L. brevis* MG5311 administration protects against ALD by suppressing CYP2E1 expression.

ALD patients appear to exhibit oxidative stress [33]. ROS increase during alcohol metabolism causes lipid peroxidation, upregulates MDA levels, and damages proteins and DNA, ultimately impairing cell function [48]. MDA is a by-product of lipid peroxidation, and the amount of MDA indirectly reflects the extent of liver damage. In a previous study, the administration of *L. plantarum* B7 and a probiotic mixture (*L. rhamnosus* L34 and *L. casei* L39) reduced MDA levels in rats with alcohol-induced liver injury [49]. *L. brevis* MG5311 administration also reduced MDA levels in the liver tissues of ethanol-fed mice.

Alcohol promotes the increased production of ROS in liver metabolism and weakens antioxidant defenses, leading to oxidative stress in the liver. ROS levels are reduced by the activation and expression of antioxidant enzymes, including SOD, CAT, and GPx. SOD is an endogenous antioxidant enzyme that catalyzes the transformation of two molecules of superoxide anions into hydrogen peroxide and oxygen. CAT catalyzes the decomposition or reduction of hydrogen peroxide to water and oxygen. GPx protects cells from oxidative stress by inhibiting lipid peroxidation in the lipid peroxidation process [50,51]. Therefore, increasing the activity of these antioxidant enzymes may be a potent strategy against ALD. Administration of a probiotic mixture (*L. plantarum* KLDS1.0344 and *L. acidophilus* KLDS1.0901) can protect against liver injury induced by 4% ethanol for 6 weeks in mice by improving the levels of SOD1, CAT, and GPx-1 [36]. MG5311 promoted SOD1 and CAT protein expression in the liver tissues in this study. It also increases the activity and expression of GPx 1/2. Thus, we have demonstrated that MG5311 administration could improve ROS-induced liver damage during alcohol metabolism by increasing the activity and expression of antioxidant enzymes.

Excessive alcohol exposure causes excessive TG accumulation in hepatocytes, interfering with liver metabolic function [31]. Representative transcription factors that regulate lipid metabolism in hepatocytes include SREBP-1c and PPARα [52,53]. SREBP-1c is a lipid synthesis transcription factor involved in cholesterol and fatty acid synthesis, and it is implicated in the pathogenesis of alcohol-induced steatosis. SREBP-1c expression is inhibited by AMPK and SIRT [54]. Activation of SIRT1 and AMPK improves hepatic steatosis by enhancing carnitine palmitoyltransferase 1 (CPT-1) expression and promoting fatty acid β-oxidation for lipolysis [55,56]. In particular, SIRT1, an NAD^+^-dependent deacetylase, can directly interact with and deacetylates SREBP-1c in an AMPK pathway-dependent or independent manner in ALD [56]. Alcohol consumption induces fatty liver by downregulating AMPK and SIRT1, increasing the acetylated form of SREBP-1c, and inhibiting fatty acid oxidation. Overexpression of SIRT1 alleviated AFLD by reducing alcohol-induced SREBP-1c hyperacetylation and activity [57]. Therefore, regulation of the SIRT1-SREBP-1c axis is one of the major mechanisms linking the pathogenesis of alcohol-induced hepatic steatosis. In this study, MG5311 administration increased SIRT1 expression and decreased SREBP-1c expression without affecting AMPK activity. Therefore, MG5311 administration inhibited fatty accumulation in the liver through SIRT1/SREBP-1c regulation in ethanol-fed mice.

Excessive alcohol exposure impairs fatty acid catabolism by inhibiting mitochondrial β-oxidation, which is the most crucial contributor to alcohol-induced fatty liver disease [52]. PPARα is a master regulator of lipid metabolism (fatty acid β-oxidation) and regulates peroxisomal catalase and cellular NAD^+^ levels during alcohol metabolism [52]. Alcohol inhibits PPARα to delay fatty acid oxidation. PPARα activity can be indirectly inhibited by the upregulation of CYP2E1-derived oxidative stress [58]. In contrast, SIRT1 positively modulates PPARα to regulate lipid homeostasis [53]. In the present study, MG5311 administration increased PPARα protein expression. These results confirmed that MG5311 improved lipid peroxidation in the liver by regulating the SIRT1/PPARα pathway.

The gut is directly linked to the liver through the portal vein (gut-liver axis). Gut microbiota dysbiosis can cause a variety of liver disorders [16,17]. Recent studies have shown that probiotics and their metabolites could improve ALD by reducing the alcohol-induced gut microbial imbalance, gut permeability, bacterial translocation, and endotoxemia, thereby restoring a balanced gut microbial community and strengthening the intestinal barrier [59,60]. This study confirmed the ameliorative effect of MG5311 on alcohol-induced oxidative stress and lipid metabolism in an ALD mouse model. Therefore, further studies are needed to confirm the direct effect of MG5311 on gut microflora and intestinal permeability.

## 5. Conclusions

**Figure 8:**
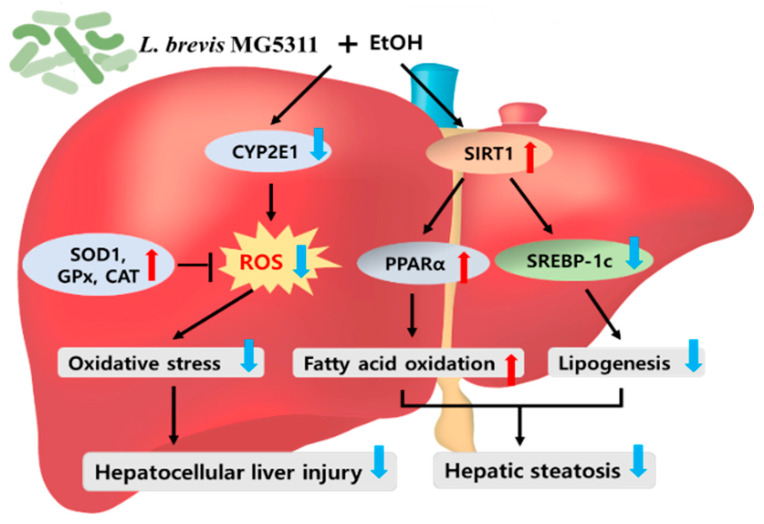
*L. brevis* MG5311 exerts hepatoprotective effects by regulating hepatic oxidative stress and lipid metabolism. CYP2E1, Cytochrome P450 2E1; SIRT1, Sirtuin 1; SOD1, superoxide dismutase 1; GPx, glutathione peroxidase; CAT, catalase; PPARα, peroxisome proliferator-activated receptor α; SREBP-1c, sterol regulatory element-binding protein 1c.

This study was conducted to evaluate whether oral administration of MG5311 has a hepatoprotective effect in ALD mice subjected to chronic plus once binge drinking. MG5311 administration to mice improved the elevated serum ALT, AST, and TG levels, which were increased by the ethanol diet. MG5311 reduced the protein expression levels of CYP2E1 and increased the protein expression levels of the antioxidant enzymes SOD1, CAT, and GPx 1/2. Moreover, MG5311 suppressed hepatic steatosis by regulating SREBP-1c and PPARα expression via upregulating the SIRT1 pathways. Taken together, it was confirmed that MG5311 administration inhibits alcohol-induced oxidative stress and promotes lipid β-oxidation in the liver (Figure 8). Therefore, MG5311 may be a potential functional probiotic candidate for preventing ALD. Further studies on the effect of MG5311 on changes in gut microbiota and intestinal permeability will be conducted.

## Data Availability

Not applicable.
